# Toxicological Effects of Tartrazine Exposure: A Review of In Vitro and Animal Studies with Human Health Implications

**DOI:** 10.3390/toxics13090771

**Published:** 2025-09-12

**Authors:** Malina Visternicu, Alexandra Săvucă, Viorica Rarinca, Vasile Burlui, Gabriel Plavan, Cătălina Ionescu, Alin Ciobica, Ioana-Miruna Balmus, Cristina Albert, Mihai Hogas

**Affiliations:** 1 Doctoral School of Biology, Faculty of Biology, “Alexandru Ioan Cuza” University of Iasi, Carol I Avenue, 20A, 700505 Iași, Romania; malina.visternicu@yahoo.ro (M.V.); rarinca_viorica@yahoo.com (V.R.); 2 “Ion Haulica” Institute, Apollonia University, Păcurari Street 11, 700511 Iași, Romania; alin.ciobica@uaic.ro; 3 Doctoral School of Geosciences, Faculty of Geography and Geology, “Alexandru Ioan Cuza” University of Iasi, Carol I Avenue, 20A, 700505 Iași, Romania; 4 Preclinical Department, Apollonia University, Păcurari Street 11, 700511 Iași, Romania; secretariat@univapollonia.ro; 5 Department of Biology, Faculty of Biology, “Alexandru Ioan Cuza” University of Iasi, Carol I Avenue, 20A, 700505 Iași, Romania; gabriel.plavan@uaic.ro (G.P.); catalinaionescu81@yahoo.com (C.I.); 6 Academy of Romanian Scientists, Splaiul Independentei Avenue No. 54, Sector 5, 050094 Bucharest, Romania; 7 CENEMED Platform for Interdisciplinary Research, “Grigore T. Popa” University of Medicine and Pharmacy, University Street, No. 16, 700115 Iași, Romania; balmus.ioanamiruna@yahoo.com; 8 Department of Exact Sciences and Natural Sciences, Institute of Interdisciplinary Research, “Alexandru Ioan Cuza” University of Iasi, Alexandru Lapusneanu Street, No. 26, 700506 Iași, Romania; 9 Physiology Department, “Grigore T. Popa” University of Medicine and Pharmacy, 700115 Iași, Romania; mihaimmh@gmail.com

**Keywords:** tartrazine, toxicity, hyperactivity, oxidative stress, genotoxicity, food additive

## Abstract

Tartrazine (TZ, also known as FD&C Yellow No. 5 or E102) is a synthetic, water-soluble yellow food dye widely used in the food and pharmaceutical industries. Some studies have associated TZ with allergic reactions, especially among people with dye sensitivities or pre-existing allergies. Recent research also suggests a possible link between TZ consumption and the worsening of behavioral disorders, especially in children, including symptoms such as hyperactivity, irritability, restlessness, and sleep disturbances. Experimental studies in laboratory animals have highlighted potential risks associated with prolonged or high-dose exposure, including toxic effects on the nervous system and liver function. In addition, increasing evidence indicates that TZ can induce oxidative stress (OS) by increasing the production of reactive oxygen species (ROS), which can contribute to cellular damage and inflammation. Although the evidence remains inconclusive, there are recommendations to limit the intake of synthetic food dyes, especially in children’s diets. The purpose of this review is to examine the potential toxic effects on health of tartrazine by analyzing findings from experimental studies in cell cultures and laboratory animals, as well as correlations observed in humans. We focus on documented adverse reactions, including possible neurotoxic, hepatotoxic, oxidative, and behavioral effects. Through this, we aim to contribute to a more comprehensive understanding of the risks associated with exposure to this synthetic food dye.

## 1. Introduction

Tartrazine is an azo dye commonly used to impart color to food products [1,2,3,4,5]. Approximately 65% of azo dyes are used as food additives and are found in various products, such as candies, jams, and soft drinks, as well as in pharmaceuticals, cosmetics, and textiles. Although TZ adds no nutritional benefit and has no nutritional value, it remains the most widely used food dye, with a significant presence in the global food industry [6,7,8,9]. According to the World Health Organization (WHO), since 2016, the acceptable daily intake (ADI) has been set at 0–10 mg/kg body weight [10]. Despite being one of the first food additives associated with potential adverse reactions, TZ continues to be approved for use in the European Union (EU) and other regions [11].

Several studies have reported an association between TZ exposure and allergic reactions such as urticaria, asthma, migraines, blurred vision, and pruritus, as well as behavioral disturbances, including hyperactivity in children [12,13,14,15,16,17]. Children appear particularly susceptible, with evidence suggesting potential links between synthetic food dyes and neurobehavioral disorders, including attention deficit/hyperactivity disorder (ADHD), as well as mutagenic or carcinogenic outcomes [18,19]. In a double-blind, placebo-controlled study, some children exposed to varying TZ doses exhibited irritability, restlessness, and sleep disturbances [20].

In recent decades, numerous experimental studies, both in vitro and in animal models, have investigated the effects of TZ on cellular functions, tissue integrity, and behavior. The results suggest this food additive may be involved in inducing OS through the generation of ROS [21,22], activating the inflammatory response through the overproduction of proinflammatory cytokines interleukin-1 (IL-1) and interleukin-6 (IL-6) [23,24], modifying enzymatic activity, and contributing to the onset of neurobehavioral disorders [25], including impairments in learning and memory processes [26,27]. From a biological mechanistic perspective, OS has emerged as a key pathway mediating TZ toxicity, contributing to an imbalance between pro-oxidants and antioxidants in cells [28,29]. This imbalance is reflected in elevated levels of malondialdehyde (MDA), a marker of lipid peroxidation, indicating increased oxidative damage. Concomitantly, endogenous antioxidant enzymes such as superoxide dismutase (SOD) and catalase (CAT) are often significantly reduced, supporting the pro-oxidant shift induced by TZ exposure [30].

However, the data available in scientific literature are heterogeneous, and translating results from preclinical experiments into the context of human exposure remains challenging. At the same time, the food industry is increasingly exploring natural alternatives to mitigate the health side effects of synthetic dyes. Therefore, replacing them with natural alternatives represents a much better option [19]. Some well-known natural alternatives include turmeric, carotenoids, annatto, saffron, and paprika extracts. For example, TZ could potentially be substituted with carotenoids extracted from pequi (*Caryocar brasiliense*) using high-performance ionic liquids, a modern, sustainable, and efficient extraction method that also enhances safety [31,32].

The aim of this review is to provide a critical and integrative analysis of experimental data from in vitro and animal model studies regarding TZ exposure, with a focus on toxicological mechanisms, including OS, neurotoxicity, and inflammatory responses. By correlating these findings with reported human side effects, this review aims to offer a comprehensive perspective on the potential risks to human health. Thus, this study contributes to clarifying a current issue regarding the safety of synthetic food additives and supports the need to reassess TZ exposure, particularly among children.

## 2. Health Implications of Tartrazine Exposure

TZ is a synthetic yellow dye widely used in food, pharmaceutical, and non-food products (Table 1). Although it improves product appearance, multiple studies have reported its potential to induce allergic reactions, asthma, or cutaneous hypersensitivity. These adverse effects have led to growing interest in natural alternatives such as annatto or β-carotene [2,25,33,34,35,36,37,38,39,40,41,42,43,44,45].

Because of its widespread use, TZ is strictly regulated. In the United States, the FDA’s Code of Federal Regulations establishes the conditions for its use in foods, drugs, and cosmetics, including batch certification and mandatory labeling to prevent hypersensitivity reactions [46]. In the EU, TZ is regulated under Commission Regulation (EC) No 1333/2006 on food additives, while its use in cosmetics is addressed by Regulation (EC) No 1223/2009 (Annex IV) [47], where it appears as trisodium 5-hydroxy-1-(4-sulphophenyl)-4-((4-sulphophenyl)azo)pyrazole-3-carboxylate and its insoluble lakes, salts, and pigments.

**Figure 1:**
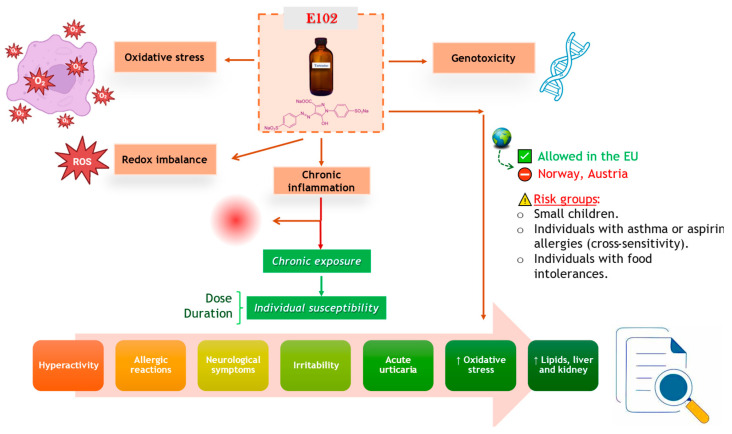
Overview of biological responses linked to Tartrazine Exposure (Figure partially created using BioRender resources (https://www.biorender.com/)).

Chemically, TZ is an anionic acid dye that converts to tartrazic acid in acidic conditions [48]. In vivo, TZ is reduced to an aromatic amine, stabilized by its nitroso derivative [49]. This compound can trigger immunological reactions, including fatigue, irritability, clinical depression, headaches, and sleep disturbances. Figure 1 summarizes the main biological pathways through which TZ may affect human health: OS, chronic inflammation, genotoxicity, and neurobehavioral disorders. These mechanisms are primarily observed under repeated or chronic exposure and may contribute to systemic conditions, especially in children and sensitive individuals. Both ingestion and dermal contact may cause hypersensitivity reactions. Some researchers suggest that symptoms may occur even at low exposure levels, with manifestations appearing up to 72 h after contact [45].

Although direct evidence linking TZ to anxiety in humans is limited, preclinical studies suggest that TZ increases OS, negatively affects liver and kidney function, alters lipid levels, and consequently raises the risk of cardiovascular disease [32,50]. Data on neurobehavioral effects are often inconsistent. The results regarding its impact on behavior and neurotoxicity are often contradictory or inconclusive.

TZ has been associated with various adverse reactions in some individuals, including migraines, agitation, asthma attacks, blurred vision, eczema, and other skin rashes. Some studies have also suggested a potentially elevated risk of thyroid cancer [49,51]. A randomized, double-blind, placebo-controlled study reported that administration of a mixture of food colorants (5 mg E110, 2.5 mg E122, 7.5 mg E102, 5 mg E124) along with 45 mg E211 was linked to negative effects on concentration, hyperactivity, and attention in children aged 3 and 8–9 years [52]. Approximately 1% of acute urticaria and/or angioedema cases were attributed to TZ [53]. Moreover, due to concerns regarding hyperactivity and behavioral effects in children, some European Member States have taken regulatory measures to ban or restrict the use of TZ. An overview of the proposed pathways and health effects associated with tartrazine exposure is presented in Figure 1.

Children with suspected hyperactivity exposed to TZ exhibited irritability, restlessness, and sleep disturbances, with severity correlating with dosage [20]. The administration of TZ in atopic patients triggered allergic reactions, predominantly cutaneous and respiratory, which were significantly more frequent compared to placebo. These included angioedema, nasal congestion, rhinorrhea, wheezing, skin rashes, and pruritus, and approximately 5% of participants showed elevated levels of TZ-specific Immunoglobulin E [54].

Data from the literature on the cytotoxic, mutagenic, and genotoxic effects of TZ remain unclear and often contradictory. Although Soares et al. [55] did not report significant cytotoxic effects, they highlighted the potential health risk of prolonged TZ exposure, suggesting it could contribute to carcinogenic processes.

Most preclinical studies demonstrating the toxic effects of TZ have used doses ranging from 100 to 700 mg/kg body weight in rodents [51,56,57,58,59,60,61,62], which is substantially higher than typical human dietary exposure (0–10 mg/kg body weight) [10]. While these high doses are useful for elucidating mechanisms of toxicity, their direct relevance to humans is limited. However, they remain important for risk assessment because cumulative exposure from multiple sources (food, pharmaceuticals, cosmetics) can approach or exceed ADI, especially in children [63]. The acceptable ADI established by JECFA is based on a NOAEL of 984 mg/kg body weight/day from chronic rat studies [10], where body weight reduction was considered the critical endpoint. While this regulatory value provides a safety margin, the in vitro and in vivo evidence summarized in this review demonstrated OS, neurotoxicity, and metabolic alteration at doses approaching or below the NOAEL.

Sensitive populations, such as individuals with allergies or metabolic disorders, may experience adverse effects even at doses below the ADI. EFSA noted that a fraction of the population may develop intolerance reactions at ADI levels [64]. The EFSA assessment took into account additional studies, including those by McCann et al. [52], which observed an increase in hyperactivity in children following the consumption of TZ-containing drinks. EFSA concluded that TZ was negative in long-term carcinogenicity studies, and the DNA migration effects observed in the in vivo Comet assay are not expected to result in carcinogenicity [64].

Children may be particularly vulnerable due to higher consumption of yellow- and orange-colored foods. Although most synthetic dyes were below the ADI, TZ was detected in several products, particularly ice cream, highlighting the need for monitoring and further research to protect this vulnerable population [65]. Taken together, high-dose animal data primarily establish hazards and mechanisms [66] but also provide biological plausibility for human health risks, reinforcing the need for prudent regulation and monitoring.

## 3. Oxidative Stress Induced by Tartrazine

Several studies in rodents have associated TZ exposure with the induction of OS [21,51,57,62,67,68] (Table 2). OS represents an imbalance between the production of ROS and the capacity of endogenous antioxidant systems to neutralize them, leading to damage to cellular components such as lipids, proteins, and DNA [69,70].

Experimental studies in Swiss white mice have shown that daily oral administration of TZ at doses as low A 2.5 and 5 mg/kg, from the first day of gestation until 15 days postnatally, induces OS in brain tissue, reflected by an increased MDA level and decreased activities of antioxidant enzymes such as glutathione (GSH) and SOD [71]. Exposure to TZ, even at concentrations considered environmentally relevant (50 mg/L), has been shown to cause significant biochemical changes, such as increased levels of MDA and nitric oxide (NO), along with a decrease in antioxidant enzyme activities (SOD, CAT, GSH) and acetylcholinesterase (AChE), suggesting neurobiochemical dysfunctions [76]. Daily administration of higher doses, such as 320 mg/kg for four weeks, exacerbates oxidative imbalance in the brain by reducing glutathione peroxidase (GPx) activity and increasing lipid peroxidation, reflected in elevated MDA levels [72]. Even significantly lower doses (7.5 mg/kg) administered over a longer period (90 days) induce severe OS, associated with increased MDA and reduced activities of endogenous antioxidant enzymes (CAT, SOD, GPx) and GSH levels [30].

Moreover, the combination of TZ and erythrosine has demonstrated synergistic effects in inducing OS in brain structures such as the striatum, where increases in MDA, AChE, and nitrites, along with reductions in GSH and CAT, suggest disruption of cerebral redox balance and a potential role in neurotoxicity [73].

Repeated exposure to TZ at doses ranging from 10–75 mg/kg over periods of up to 8 weeks has been associated with marked alterations in oxidative balance in key organs such as the liver, kidneys, and pancreas. This occurs through decreased levels of endogenous antioxidants (GSH, SOD, CAT, glutathione reductase (GR)) and increased MDA, reflecting a significant contribution to metabolic and endocrine dysfunctions [1,74,75].

Additional studies have shown that high doses (200–500 mg/kg) exacerbate systemic OS, affecting the liver, kidneys, spleen, and blood. Marked increases in MDA [29,56,57,59,61,62] were observed, along with decreases in GSH, CAT, and SOD [29,56,57,59], TOS, and TAS [59], as well as alterations in histopathological parameters [29,56,57,59,62]. However, Golli et al. [62] reported increases in CAT and GST levels. These imbalances are correlated with marked histopathological changes in the liver, kidneys, and intestines, supporting the idea of systemic toxicity via oxidative mechanisms.

Also, the studies by Amin et al. [68] and Gao et al. [51] highlight the impact of doses ranging from 15 to 700 mg/kg on the liver and brain through the inhibition of GSH, SOD, CAT and the increase in lipid peroxidation [51,68], reflecting the impairment of cognitive functions and potential neurotoxicity. These effects are attributed to the formation of free radicals during the metabolism of TZ, which promotes the activation of inflammatory pathways and increased expression of IL-1 and IL-6, reinforcing the role of chronic inflammation in the toxicity of this substance [51].

Taken together, these studies indicate that TZ disrupts redox homeostasis by increasing ROS and depleting endogenous antioxidants such as SOD, CAT, GPx, and GSH, leading to lipid peroxidation, protein oxidation, and protein DNA damage [1,29,30,51,57,61,67,68,70,72,77,78,79,80]. This oxidative imbalance contributes to neurotoxicity, hepatotoxicity, and systemic organ dysfunction and may interact synergistically with other food additives such as erythrosine. While most data derive from rodent models at doses above human ADI, this finding provides mechanistic insight into potential human health effects, especially in sensitive populations [1,51,56,57]. The close link between OS, inflammation, and metabolic or neurobehavioral alteration underscores the biological plausibility of adverse outcomes in humans [51,68].

## 4. In Vitro Studies on Tartrazine Toxicity

In vitro experiments provide a controlled environment to assess the direct cellular effects of TZ and to explore potential molecular mechanisms of toxicity. These studies complement in vitro findings and help identify specific cell type susceptibilities. Several in vitro studies have assessed the cytotoxic and genotoxic effects of TZ in different types of human cells and experimental models (Table 3). In human lymphocytes exposed to concentrations ranging from 0.25 to 64.0 mM, TZ did not show cytotoxicity but induced significant genotoxic effects at all tested concentrations, with only partial DNA repair observed [55]. Similarly, human leukocytes exposed to TZ at 5–500 μg/mL showed no cytotoxic or mutagenic effects; however, DNA damage was observed at concentrations ≥70 μg/mL, consistent with in silico toxicity predictions [77].

Taken together, in vitro studies demonstrated that TZ exhibits low cytotoxicity in normal human cells such as lymphocytes [55,80] and fibroblasts [78], even at high concentrations, but consistently induces genotoxic alterations in lymphocytes [55,77,80] and epigenetic modifications in keratinocytes and cancer cell lines [81]. These findings suggest interference with DNA integrity and chromatin regulation. TZ induces stronger cytotoxic effects in some cancer cell types, indicating differential susceptibility depending on cell type. Mechanistically, these effects may involve induction of OS [78], disruption of the DNA repair pathway [55,77], and modulation of epigenetic regulations such as DNA methyltransferases and histone deacetylases [81]. The moderate estrogenic activity observed in breast cancer cells further suggests that TZ could interact with hormonal pathways under certain conditions [79]. 

An in vitro study on human foreskin fibroblasts evaluated the effects of several dyes, including TZ. Testing revealed that TZ did not induce significant cytotoxic effects, even at high concentrations. In contrast, indigo carmine and chlorophyllin exhibited marked cytotoxicity at elevated doses. Moreover, chlorophyllin also triggered increased ROS production, indicating potential OS. These findings underscore the importance of further research into the safety of dyes commonly used in pharmaceutical and cosmetic formulations [78].

Investigations into the estrogenic, cytotoxic, and genotoxic potential of TZ compared to phytoestrogens using yeast assays and MCF-7 breast cancer cells demonstrated that TZ possesses moderate estrogenic activity, with low cytotoxicity and no genotoxicity observed in short-term exposures [79]. Additionally, TZ tested at concentrations of 2.5, 5, and 10 mM showed no genotoxicity and minimal cytotoxicity in human lymphocytes but induced significant cytotoxic effects in melanoma cells [80]. Exposure to TZ was associated with upregulation of enzymes involved in epigenetic regulation in human cells such as HaCaT, HepG2, and A549, which may influence cell proliferation and survival, potentially favoring activation of oncogenic pathways. Furthermore, significant DNA fragmentation was observed, indicating a potential genotoxic effect. These results suggest that TZ may contribute to epigenetic disruptions and increased cancer risk [81].

Although the concentrations used in vitro are generally higher than typical human dietary exposure, these findings provide biological plausibility for genotoxic and epigenetic effects. Overall, in vitro evidence indicates that, while TZ has limited cytotoxicity in normal human cells, it can induce genotoxic effects and notable cytotoxicity in certain cancer cell lines. Differences in responses between cell types highlight the need to use a variety of cell models when evaluating the safety of this widely used azo dye.

## 5. Tartrazine Toxicity in Experimental Animal Models

Experimental studies in animal models indicate that TZ exerts multisystem toxicity, particularly under chronic or high-dose exposure (Table 4). Data obtained from *Danio rerio* show increased sensitivity to TZ during embryonic development. According to the study by Joshi and Katti [82], exposure to concentrations ≥10 mM was associated with the occurrence of deformities, edema, cardiovascular dysfunctions, delayed hatching, and increased mortality. At concentrations ≥75 mM, embryonic development was completely inhibited, highlighting the significant toxic potential of the substance on embryonic development. Similar findings were reported by Jiang et al. [83], who observed reduced embryonic survival, delayed hatching, swelling of the heart, and deformities along the body axis, with estimated effective concentration for 50% of the population and lethal concentration for 50% of the population values of 42.66 mM and 47.10 mM, respectively. Gupta et al. [84] further noted that exposure to TZ in embryonic water, especially at concentrations of 0.5% and above, significantly increased hatching rates and altered SOD1 gene expression during early developmental stages. Additionally, Thanh et al. [85] identified a vascular toxicity profile manifested by hemorrhages, edema, and abnormal vessel branching, correlated with altered migration and proliferation of endothelial cells. Adverse effects were also observed under chronic exposure conditions. Linskens et al. [86] demonstrated that prolonged administration of TZ (22 μM) caused cognitive deficits in adult fish, reflected by decreased learning capacity and cognitive flexibility. These changes were not present with limited exposure during the early post-embryonic period, suggesting a duration-dependent manifestation of neurotoxicity.

In rodents, oral administration of TZ induces significant structural and functional changes. Meena and Meena [87] reported that TZ caused a dose-dependent increase in body weight, deformation of seminiferous tubules, decreased Leydig and Sertoli cell numbers, and reduced spermatozoa. Similarly, Arefin et al. [88] reported evidence of hepatotoxicity and nephrotoxicity at doses of 200–400 mg/kg, including significant increases in bilirubin, creatinine, and body weight. TZ significantly affects glucose homeostasis and pancreatic endocrine function, being associated with increased blood glucose levels and lipase activity, as well as decreased insulin, calcium, and magnesium levels after 30 days of administration [1]. Other histopathological effects include dilation of perineural spaces and severe degeneration of Purkinje cells, edema, and increased astrocyte populations. Subchronic exposure to TZ has been correlated with alterations in hepatic and renal parameters, evidenced by increases in alanine aminotransferase (ALT), aspartate aminotransferase (AST), urea, and total protein [61], as well as transaminases, lactate dehydrogenase (LDH), creatinine, and uric acid, with decreases in total proteins, albumin, and globulins [51,72,73].

At the neurobehavioral level, TZ induces hyperactivity, anxiety, and cognitive deficits in behavioral tests, alongside cerebral histological changes, including neuronal apoptosis and vascular congestion, highlighting its neurotoxic effect [51,72,73]. In a complementary study, Kamel and El-Lathey [89] reported anxious and depressive effects, as well as decreased social interaction in male Wistar rats, suggesting a cumulative negative impact on mental health.

Chronic exposure (7.5–500 mg/kg) has been correlated with increases in hepatic enzymes (ALT, AST, alkaline phosphatase (ALP)) [26,30,56,60,68,74,90,91], renal markers (urea, creatinine, uric acid) [56,60,74,90,91], and lipid parameters (low-density lipoprotein (LDL), triglycerides) [30,59,74,90,91]. These changes were accompanied by histopathological lesions in vital organs such as the liver, kidneys, testes, stomach, colon, heart, and central nervous system [30,56,57,74,90,91,92,93,94]. Hematological and immunological alterations included leukocytosis, increased tumor necrosis factor alpha (TNF-α), and splenic changes, alongside decreases in hemoglobin and platelets [93,95]. Endocrine disorders, electrolyte imbalances, and neuroinflammation were also reported [73].

Altinoz et al. [57] reported severe intestinal lesions characterized by reduced intestinal antioxidant levels and increases in lipid peroxidation. Additionally, cardiovascular impairment was suggested by elevated cardiac troponin, creatine kinase, and non-high-density lipoprotein (HDL) cholesterol [92], parameters commonly used as early indicators of myocardial injury, suggesting a potential cardiovascular risk under prolonged exposure conditions.

A significant number of studies have investigated the protective role of natural compounds. Among these, Curcumin, crocin, Nigella sativa oil, chlorophyll, and spinach fruit extract have proven effective in reducing OS, normalizing biochemical parameters, and alleviating tissue damage [30,56,57,58,59,74,75,91,93,94,97]. Al-Seeni et al. [74] reported that administration of 10 mg/kg for 8 weeks resulted hepatic and renal dysfunctions, histopathological lesions, and alterations in lipid profile and oxidative markers, with partial improvement following administration of *Nigella sativa* oil. Comparatively, natural yellow coloring agents such as curcumin and β-carotene not only provide coloration but also exhibit antioxidant and anti-inflammatory properties [98,99,100,101,102], highlighting their potential as a safer alternative to TZ. For instance, curcumin and β-carotene have been shown in multiple studies to exhibit antioxidant and anti-inflammatory effects, with curcumin reducing OS and inflammation in cell and animal models [98,99,100], and β-carotene potentially modulating pathways such as TNF and sphingomyelin signaling [101,102]. Integrating these natural compounds into foods could mitigate the risks associated with synthetic azo dyes while maintaining product appeal.

Available data consistently highlights the systemic toxic effects of TZ, manifested through hepatic, renal, hematological, metabolic, and neurological dysfunctions, confirmed by both biochemical changes and histopathological lesions. Prolonged exposure also induces hematological and immunological disorders, disturbances in glucose and electrolyte metabolism, and negative effects on the central nervous system, including anxiety, depression, reduced locomotor activity, and neuroinflammation [30,56,73,90,93,95,96].

## 6. Conclusions and Future Perspectives

Tartrazine remains a widely used synthetic food additive; yet, accumulating scientific evidence raises serious concerns about its safety. Preclinical studies consistently report toxic effects such as oxidative stress, inflammation, neurotoxicity, metabolic disruption, hepatotoxicity, and potential genotoxicity. Even though these findings, such as antioxidant system impairment and activation of inflammatory pathways are mostly from animal models, these results are relevant to human health. Although direct clinical evidence is limited, epidemiological data suggests associations between tartrazine exposure and adverse effects like hyperactivity, allergies, migraines, and behavioral issues, particularly in children. Considering the potential for cumulative exposure, individual variability, and unclear thresholds for chronic toxicity, a re-evaluation of tartrazine’s safety is both timely and necessary. Replacing tartrazine with safer, natural alternatives should be prioritized to protect public health, especially for sensitive populations. To better understand the risks associated with tartrazine, future studies should focus on long-term clinical investigation, the identification of specific biomarkers, and clarification of the epigenetic mechanisms involved. Simultaneously, developing safe natural alternatives and reassessing current regulations remain essential priorities to ensure public health protection.

## Figures and Tables

**Table 1:** Common Uses of Tartrazine and Relevant Toxicological Concerns.

Category	Product Examples	References
Food products	TZ is widely used to impart an intense yellow color in various food products such as bread, beverages, cereals, peanuts, candies, jellies, chewing gum, flavored chips, creams, ice cream, yogurts, cakes, instant desserts, soups, sauces, jams, flavored rice, and pasta. Due to potential adverse effects, there is a growing tendency to replace it with natural pigments like annatto or β-carotene.	[2,35,37,40,41,42,43,45]
Pharmaceuticals	TZ is used as a coloring excipient in multivitamins, gelatin capsules, tablets, syrups, and pediatric medicines. In sensitive individuals, it may cause allergic reactions or asthma.	[33,36,38,39,40]
Non-food products	TZ is also present in non-food products such as soaps, cosmetics, shampoos, hair conditioners, pastels, crayons, and stamp dyes. Skin contact may cause hypersensitivity reactions.	[25,34,44,45]

TZ—Tartrazine.

**Table 2:** Evidence of Oxidative Imbalance Following Tartrazine Exposure in Rodent Models.

Experimental Organism	*n*=	Species/Strain	Age/Weight	Dose	Time	Method of Administration	Sample	Effect	Ref.
Mice	60	Swiss white mice	25–30 g	2.5 and 5 mg/kg	day one of pregnancy to day 15 after birth.	oral gavage	Brain tissue	↑ MDA ↓ GSH ↓ SOD	[71]
Rats	18	Young male albino rats	28 days, 60–80 g	320 mg/kg tartrazine in 1 mL distilled water, daily	4 weeks	oral gavage	Brain	↓ GPx ↑ MDA	[72]
	50	Wistar male albino rats	180 and 200 g	7.5 mg/kg	90 days	diets containing dry mass	Liver	↑ MDA ↓ GSH	[30]
							Serum	↓ SOD ↓ CAT ↓ GPx	
	24	Male Wistar rats	10–12 weeks, 180–200 g	2, 6, 10 mg/kg (erythrosine + TZ 50:50 mix)	6 weeks	oral gavage	Brain tissue	↑ MDA ↓ GSH ↓ CAT ↑ ACHe	[73]
	18	Male albino rats	175–185 g	10 mg/kg (+3.75 mg/kg sulfanilic acid)	8 weeks	oral administration	Serum, liver, and kidney tissue homogenate	↑ MDA ↓ GSH ↓ SOD ↓ CAT ↓ GR	[74]
	18	White albino rats	-	Low (10 mg/kg) and high (50 mg/kg) doses	15 and 30 days	oral administration	Serum	↓ SOD	[1]
	30	Sprague–Dawley male albino rats	150–200 g	75 mg/kg	90 days	oral administration by orogastric gavage	Hepatic and renal tissue homogenate	↑ MDA ↓ GSH ↓ SOD ↓ CAT	[75]
	18	Male albino rats	10–15 weeks, 190–250 g	400 mg/kg	30 days	oral administration	Serum	↑ MDA ↓ GSH ↓ SOD ↓ CAT ↓ GPx	[29]
	40	Female Wistar albino rats	225–250 g	500 mg/kg	21 days	oral gavage	Tissue homogen ates	↑ MDA ↑ SOD ↑ TOS ↓ GSH ↓ CAT ↓ TAS	[56]
	40	Adult female Wistar rats	225–250 g, 8–10 weeks	500 mg/kg	3 weeks	oral gavage	Tissue homogenates	↑ MDA ↑ TOS ↓ GSH ↓ SOD ↓ CAT ↓ TAS	[57]
	30	Adult male Sprague–Dawley rats	120–150 g	200 mg/kg	60 days	oral administration	Tissue homogenate	↑ MDA	[61]
	40	Female Wistar rats	225–250 g	500 mg/kg	3 weeks	oral gavage	Tissue homogenate	↑ MDA ↑ TOS ↓ GSH ↓ TAS ↓ SOD ↓ CAT	[59]
	20	Male Wistar rats	130 ± 40 g	300 mg	30 days	oral administration	Tissue homogenate	↑ MDA ↑ CAT ↑ GST	[62]
	36	Young male albino rats	60–80 g	low doses of TZ 15 mg/kg bw	30 days	oral administration	Liver tissue homogenate	↑ MDA ↓ CAT ↓ SOD	[68]
				high doses were 500 mg/kg bw				↑ MDA ↓ CAT ↓ SOD ↓ GSH	
	40	Sprague–Dawley rats	70 ± 10 g	0, 175, 350, and 700 mg/kg bw	30 days	oral gavage	Brain tissue	↓ GSH ↓ SOD ↑ MDA	[51]

ACHe—Acetylcholinesterase; bw—Body Weight; CAT—Catalase; GPx—Glutathione peroxidase; GR—Glutathione reductase; GSH—Glutathione; GST—Glutathione S-transferase; MDA—Malondialdehyde; SOD—Superoxide dismutase; TAS—Total Antioxidant Status; TOS—Total Oxidant Status; TZ—Tartrazine, ↑—significantly increased; ↓—significantly decreased.

**Table 3:** Summary of Experimental Studies Assessing the Cytotoxic and Genotoxic Effects of Tartrazine in Different Human Cell Types and Assays.

Cell Type	Concentration Tested	Tests Performed	Key Findings	Ref.
Human lymphocytes	0.25–64.0 mM	MTT assay, alkaline comet assay	No cytotoxicity; genotoxic at all doses; partial DNA repair.	[55]
Human leukocytes	5–500 μg/mL	Trypan Blue viability, Micronucleus test, Comet assay, Cytogenetics, In silico	No cytotoxicity/mutagenicity; DNA damage at ≥70 μg/mL; supported by in silico models.	[77]
Human foreskin fibroblasts	10, 100, 250, 500, 1000, and 2000 μg/mL for various dyes	MTT assay, ROS, lipid peroxidation, LDH	TZ: no effect; indigo carmine/chlorophyllin cytotoxic at high doses	[78]
Yeast assay, MCF-7 breast cancer cells	Not specified (short-term)	Estrogenic activity, LDH release, micronucleus test	8-PN showed the strongest estrogenic effect, followed by TZ and genistein; all exhibited low cytotoxicity and no genotoxicity.	[79]
Human lymphocytes, GR-M melanoma cells	2.5, 5, and 10 mM	Chromosome aberration, CBMN assay, trypan blue test	No genotoxicity in lymphocytes; low cytotoxicity in lymphocytes; high cytotoxicity in melanoma cells	[80]
HaCaT	20 µM, 40 µM, and 80 µM	qRT-PCR Alkaline Comet Assay	Upregulated DNMT and HDAC genes with increased DNA fragmentation, indicating epigenetic and genotoxic effects.	[81]
HepG2				
A549				

8-PN—8-Prenylnaringenin; CBMN assay—Cytokinesis-Block Micronucleus Assay; DNA—Deoxyribonucleic Acid; DNMT—DNA Methyltransferase; HaCaT—immortalized human keratinocyte; HDAC—Histone Deacetylase; HepG2—Human liver cancer cell line; LDH—Lactate Dehydrogenase; MTT—Methyl Thiazolyl Tetrazolium; qRT-PCR—Quantitative Real-Time Polymerase Chain Reaction; ROS—reactive oxygen species; TZ—Tartrazine.

**Table 4:** Effects of Tartrazine on Behavior and Biochemistry in Animal Models.

Experimental Model	Number (*n*=)	Method of Administration	Time	Dose	Analysis	Effect	Ref.
Zebrafish							
Zebrafish embryo	280 (20/concentration)	Exposed to E3 medium with varying TZ concentrations in Petri dishes	24, 48, 72, 96, 120, 144, 168 hpf	0, 0.1, 1, 2, 3, 4, 5, 10, 20, 30, 40, 50, 75, 100 mM	Developmental anomalies (heart rate, edema, tail distortion, hatching, mortality) observed via bright field microscopy.	Control embryos hatched normally; ≥10 mM caused early hatching with deformities and ≥40 mM increased mortality.	[82]
	25 embryos/well	Embryos exposed in 6-well plates with E3 medium supplemented with TZ	3–4 h post-fertilization to 4 dpf	0, 5, 10, 20, 50 g/L	Zebrafish embryo toxicity and vascular defects.	Dose-dependent vascular defects: hemorrhage, edema, small eye, vessel abnormalities.	[85]
	20 embryos/well	Exposed in E3 medium	72 h (hpf)	5–100 mM (various concentrations)	Developmental and cardiac toxicity parameters	TZ caused dose-dependent drops in survival, hatching, cardiac/yolk sac edema, spinal defects, and heart rate.	[83]
	9	Exposure via aquatic media	6 months to a year	22 μM	Behavioral tests: T-maze test, cognitive flexibility, memory, learning, perseverance, consistency in choices.	Learning, memory, and flexibility impaired task completion and perseverance reduced.	[86]
	100	Exposed to varying erythrosine and TZ levels in embryo water.	Up to 10 dpf	Erythrosine: 0.001–0.1%; TZ: 0.01–0.5%	Biochemical and genetic analyses	High TZ (≥0.5%) boosted hatching (55% at 48 hpf, 100% at 72 hpf) and triggered SOD1 expression via OS.	[84]
Mice							
KunMing mouse (20 ± 2 g)	40	Oral gavage	30 days	0, 175, 350, and mg/kg body mass	Behavioral (Step-through, Morris maze) and biochemical tests	TZ negatively affects learning and memory in mice, increasing escape time and reducing reaction time in tests.	[51]
Male Swiss albino mice (4 weeks)	15	Oral administration	72 days	100 and 200 mg/kg	Histological analyses	Increased bw, mild deformation of seminiferous tubules, moderate reduction of Leydig and Sertoli cells, fewer spermatozoa	[87]
Swiss albino mice (25–30 g)	15	Oral administration	25 days	200 mg/kg	Physiological and biochemical analyses	Insignificant decrease in the cholesterol level, no significant in triglyceride, significantly increased bilirubin and creatinine	[88]
				400 mg/kg		Insignificant decrease in the cholesterol level, significant increase in triglycerides, bilirubin, and creatinine	
Rats							
Sprague–Dawley rats (70 ± 10 g)	40	Oral gavage	30 days	175, 350, 700 mg/kg body mass	Behavioral tests: Open-field test. Biochemical analyses	TZ increases activity and anxiety in rats, also causing histopathological changes in the brain.	[51]
Male Wistar rats (40–50 g)	45	Dissolved in tap drinking water	16 weeks	0%, 1% (low dose) and 2.5% (high dose)	Behavioral tests: Open field behaviour test Elevated plus maze test Light-Dark transition task Forced swim test Social interaction test	The study highlights the harmful effects of TZ on anxiety and depression, highlighting the risks of long-term exposure to food dyes on mental health.	[89]
Young male albino rats (28 days old, 60–80 g)	18	Oral gavage	4 weeks	320 mg/kg TZ in 1 mL distilled water, once daily.	Neurobiological and histological analysis: Brains were harvested and analyzed for histological changes.	TZ has a neurotoxic effect, evidenced by histological changes such as neuronal apoptosis and vascular congestion.	[72]
White albino rats of either sex	18	Oral administration	15 and 30 days	Low dose: 10 mg/kg High dose: 50 mg/kg	Biochemical, hormonal, and histological analyses	TZ disrupts glucose balance, damages pancreas, alters endocrine function. Increases glucose, lipase decreases insulin, Ca, Mg	[1]
Male albino rats	18	Oral administration	8 weeks	10 mg/kg (+3.75 mg/kg sulfanilic acid)	Biochemical and histological	It caused liver and kidney dysfunction with lesions. Increased cholesterol, triglycerides, LDL, VLDL, ALT, AST, ALP, bilirubin, creatinine, urea, uric acid. Decreased HDL, total protein.	[74]
Wistar male albino rats	50	Diets containing dry mass	90 days	7.5 mg/kg	Biochemical and histological analyses	TZ raised lipids, liver enzymes, kidney function. Increased total cholesterol, triglycerides, LDL, ALT, AST, ALP, LDH.	[30]
Female Wistar albino rats (225–250 g)	40	Oral gavage	21 days	500 mg/kg	Biochemical analyses and histopathological examinations	Increased AST, ALT, ALP indicating liver damage.	[56]
Male albino rats (65–80 g)	12	Oral administration	7 weeks	7.5 and 75 mg/kg	Biochemical and histopathological analyses	Study showed harmful lipids, biochemical changes, and liver/kidney damage. Increased cholesterol, triglycerides, LDL, VLDL, ALT, AST, ALP, creatinine, urea, uric acid.	[90]
Male Wistar albino rats (200–250 g)	40	Oral administration	50 days	7.5 mg/kg	Biochemical and histopathological analyses	TZ impaired liver/kidney, altered histology, lipids, glucose. Increased ALT, AST, ALP, GGT, urea, uric acid, creatinine, protein, cholesterol, triglycerides, LDL decreased HDL.	[91]
Adult female Wistar rats (225–250 g, 8–10 weeks old)	40	Oral gavage	3 weeks	500 mg/kg	Biochemical and histopathological analyses	TZ caused degenerative and metaplastic changes in ileum and colon epithelium.	[57]
Wistar albino rats (146–153 g)	20	Dissolved in 1 mL of distilled water	30 days	7.5 mg/kg bw	Biochemical, histological, and ultrastructural analyses	TZ raised AST, ALT, ALP, uric acid, urea, creatinine, reduced antioxidants, and caused liver and kidney damage.	[26]
Male Wistar rats (10–12 weeks old, 180–200 g)	24	Oral gavage	6 weeks	2, 6, 10 mg/kg (50:50 erythrosine-TZ)	Behavioral (open field test, forced swimming test, tail suspension test), biochemical and enzymatic analyses	Increased nitrite, TNF-α worsened anxiety and depression.	[73]
Sprague–Dawley male albino rats (150–200 g)	30	Oral administration by orogastric gavage	90 days	75 mg/kg	Biochemical, genetic, immunohistochemical, histology analyses	Increased AST, ALT, urea, creatinine liver and kidney damage.	[75]
Female Wistar albino rats (225–250 g)	40	Oral gavage	21 days	500 mg/kg	Biochemical and histopathological analyses	TZ caused kidney glomerular collapse, inflammation, congestion.	[58]
Albino rats (~0.2 kg)	63	Oral administration	30 and 60 days	7.5 mg/kg	Biochemical and histopathological analyses	TZ damages heart, raises nHDL and creatine kinase, increasing cardiovascular risk.	[92]
Male rats (10–15 weeks old, 190–250 g)	18	Oral administration	30 days	400 mg/kg	Biochemical analyses	Increased ALT, AST, ALP, urea, uric acid, creatinine decreased Na, K, Ca.	[60]
Adult male Sprague–Dawley albino rats	30	Oral administration	90 days	1.35 mg/kg	Hematological, immunological, and histopathological analyses	Decreased hemoglobin, RBC, PCV%, platelets increased WBC, neutrophils, lymphocytes, monocytes.	[93]
Female Wistar rats (225–250 g)	40	Oral gavage	3 weeks	500 mg/kg	Biochemical and histopathological analyses	Increased total cholesterol, glucose, triglycerides, LDL, VLDL decreased HDL.	[59]
Albino Wistar rats	20	Oral administration	7 weeks	75 mg/250 mL water 100 mg/250 mL water	Biochemical, hematological, and histopathological analyses	TZ damaged liver, kidneys, spleen no change in cholesterol, triglycerides, ALT. Increased AST, creatinine, WBC, neutrophils, lymphocytes.	[94]
Adult male Sprague–Dawley rats (120–150 g)	30	Oral administration	60 days	200 mg/kg	Biochemical, histological, and physiological analyses	Subchronic TZ affects liver and kidney parameters and induces OS. Increased ALT, AST, urea, total protein.	[61]
Male and female Wistar rats (170–200 g)	30	Oral administration	13 weeks	5 mg/kg	Hematological and histopathological analyses	No effect	[95]
				7.5 mg/kg		Decreased platelets increased neutrophils, basophils, and mean platelet volume.	
				10 mg/kg		No effect	
Adult male albino rats (120–150 g)	40	Oral gavage	30 days	7.5 mg/kg bw	Histopathological and Immunohistochemical analyses	TZ causes structural damage in cerebellum, glands, kidneys, with edema, congestion, neuron vacuolization, and cell deformation.	[96]
				15 mg/kg bw		Edema, dilated perineural spaces, and degenerating Purkinje cells.	
				100 mg/kg bw		Severe Purkinje cell degeneration, gray matter vacuolization, edema, nuclear pyknosis, vessel engorgement, increased astrocytes	
Male Wistar rats (130 ± 40 g)	20	Oral administration	30 days	300 mg	Biochemical and histopathological analyses	Increased transaminases, LDH, creatinine, uric acid, kidney proteins decreased total protein, albumin, globulin HDL unchanged.	[62]
Young male albino rats (*Rattus norvegicus*), 60–80 g	36	Oral administration	30 days	Low dose: 15 mg/kg bw	Biochemical analyses	Increased ALT, AST, ALP, total protein, albumin, globulin, creatinine, urea decreased serum cholesterol.	[68]
				High dose: 500 mg/kg bw		Increased ALP, total protein, albumin, creatinine, urea	

ALP—Alkaline Phosphatase; ALT—Alanine Aminotransferase; AST—Aspartate Aminotransferase; bw—Body weight; Ca—Calcium; dpf—Days Post Fertilization, GGT—Gamma-Glutamyl Transferase; HDL—High-Density Lipoprotein; hpf—Hours Post Fertilization; LDH—Lactate Dehydrogenase; LDL—Low-Density Lipoprotein; MPV—Mean Platelet Volume; Na—Sodium; OS—Oxidative Stress; PCV%—Packed Cell Volume Percentage (hematocrit); RBC—Red Blood Cells; SOD1—Superoxide Dismutase 1; TNF-α—Tumor Necrosis Factor-alpha; TZ—Tartrazine; VLDL—Very Low-Density Lipoprotein; WBC—White Blood Cells.

## Data Availability

No new data were created or analyzed in this study. Data sharing is not applicable to this article.
